# 2-Nitro-*N*-(8-quinolyl)benzamide

**DOI:** 10.1107/S160053680803777X

**Published:** 2008-11-20

**Authors:** Gang Lei, Lin-Hai Jing, Li Zhou

**Affiliations:** a School of Chemistry and Chemical Engineering, China West Normal University, Nanchong 637002, People’s Republic of China

## Abstract

In the title compound, C_16_H_11_N_3_O_3_, the amide group is twisted away from the quinoline ring system and nitro­benzene ring by 8.02 (1)° and 54.92 (1)°, respectively. The crystal packing is stabilized by inter­molecular C—H⋯O hydrogen bonds, and π–π inter­actions between the quinoline ring systems of inversion-related mol­ecules, with a centroid–centroid distance of 3.4802 (13) Å.

## Related literature

For the biological activities of quinoline derivatives, see: Oku *et al.* (1998 ▶, 1999 ▶).
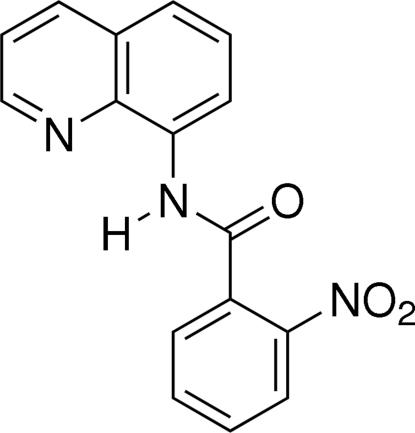


## Experimental

### 

#### Crystal data

C_16_H_11_N_3_O_3_
*M* *_r_* = 293.28
Monoclinic, 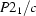

*a* = 12.430 (3) Å
*b* = 10.144 (3) Å
*c* = 11.528 (3) Å
β = 116.449 (3)°
*V* = 1301.4 (6) Å^3^
*Z* = 4
Mo *K*α radiation
μ = 0.11 mm^−1^
*T* = 93 (2) K
0.50 × 0.40 × 0.15 mm

#### Data collection

Rigaku R-AXIS RAPID diffractometer
Absorption correction: none
10410 measured reflections
2949 independent reflections
2679 reflections with *I* > 2σ(*I*)
*R*_int_ = 0.029

#### Refinement

*R*[*F* ^2^ > 2σ(*F* ^2^)] = 0.051
*wR*(*F* ^2^) = 0.145
*S* = 1.00
2949 reflections
199 parameters
H-atom parameters constrained
Δρ_max_ = 0.22 e Å^−3^
Δρ_min_ = −0.28 e Å^−3^

### 

Data collection: *RAPID-AUTO* (Rigaku, 2004 ▶); cell refinement: *RAPID-AUTO*; data reduction: *RAPID-AUTO*; program(s) used to solve structure: *SHELXS97* (Sheldrick, 2008 ▶); program(s) used to refine structure: *SHELXL97* (Sheldrick, 2008 ▶); molecular graphics: *XP* in *SHELXTL* (Sheldrick, 2008 ▶); software used to prepare material for publication: *SHELXL97*.

## Supplementary Material

Crystal structure: contains datablocks global, I. DOI: 10.1107/S160053680803777X/ci2716sup1.cif

Structure factors: contains datablocks I. DOI: 10.1107/S160053680803777X/ci2716Isup2.hkl

Additional supplementary materials:  crystallographic information; 3D view; checkCIF report

## Figures and Tables

**Table 1:** Hydrogen-bond geometry (Å, °)

*D*—H⋯*A*	*D*—H	H⋯*A*	*D*⋯*A*	*D*—H⋯*A*
C3—H3⋯O3^i^	0.95	2.55	3.209 (2)	127
C4—H4⋯O2^ii^	0.95	2.48	3.319 (2)	147
C17—H17⋯O1^iii^	0.95	2.42	3.160 (2)	135

Symmetry codes: (i) 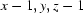; (ii) 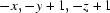; (iii) 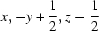.

**Fig. 1.**
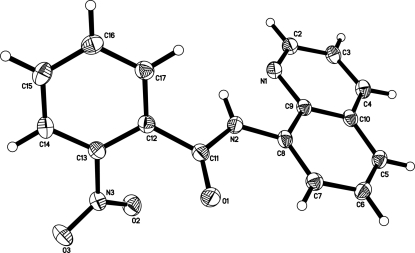
The molecular structure of the title compound, showing 50% probability displacement ellipsoids and the atomic numbering.

## supplementary crystallographic information

### Comment

Quinoline derivatives are a class of important compound for the treatment of bone metabolic disorders (Oku *et al.*,  1998) and as H^+^-ATPases inhibitors (Oku *et al.*,  1999). We report here the crystal structure of the title compound.

Bond lengths and angles in title molecule (Fig.1) are normal. The quinoline ring system is planar, with a maximum deviation of 0.033 (2) Å for atom C3. As a result of steric effects, the amide group is twisted away from the planes of the quinoline ring system and the nitrobenzene ring. The C5-C10 and C12-C17 planes form dihedral angles of 8.02 (1) and 54.92 (1)°, respectively, with the O1/N2/C8/C11 plane. The dihedral angle between the C12-C17 and O2/O3/N3/C13 planes is 36.83 (1)°.

The crystal packing is stabilized by C—H···O hydrogen bonds (Table 1), and π-π interactions between the benzene rings of the inversion-related molecules at (*x*, *y*, *z*) and (-*x*, 1 - *y*, 1 - *z*), with a centroid-centroid distance of 3.4802 (13) Å.

### Experimental

*O*-Nitrobenzoic acid (2 mmol) and an excess of thionyl chloride (3 mmol) in dioxane (20 ml) were boiled under reflux for 6 h. The solution was distilled under reduced pressure and a yellow solid was obtained. 8-Aminoquinoline (2 mmol) in tetrahydrofuran (20 ml) was added to the yellow solid and boiled under reflux for 6 h. The solution was then cooled to ambient temperature and filtered to remove the tetrahydrofuran. The precipitate was dissolved in the dimethyl sulfoxide and allowed to stand for one month at ambient temperature, after which time white single crystals of the title compound suitable for X-ray diffraction were obtained.

### Refinement

All H atoms were placed in calculated positions, with C-H = 0.95 Å and N-H = 0.88 Å, and refined using a riding model, with *U*_iso_(H) = 1.2*U*_eq_(C,N).

### Crystal data

**Table:**

C_16_H_11_N_3_O_3_	*F*_000_ = 608
*M**_r_* = 293.28	*D*_x_ = 1.497 Mg m^−^^3^
Monoclinic, *P*2_1_/*c*	Mo *K*α radiation λ = 0.71073 Å
Hall symbol: -P 2ybc	Cell parameters from 3984 reflections
*a* = 12.430 (3) Å	θ = 3.2–27.5º
*b* = 10.144 (3) Å	µ = 0.11 mm^−^^1^
*c* = 11.528 (3) Å	*T* = 93 (2) K
β = 116.449 (3)º	Block, white
*V* = 1301.4 (6) Å^3^	0.50 × 0.40 × 0.15 mm
*Z* = 4	

### Data collection

**Table:**

Rigaku R-AXIS RAPID diffractometer	2679 reflections with *I* > 2σ(*I*)
Radiation source: fine-focus sealed tube	*R*_int_ = 0.029
Monochromator: graphite	θ_max_ = 27.5º
*T* = 93(2) K	θ_min_ = 3.5º
ω scans	*h* = −16→16
Absorption correction: none	*k* = −12→13
10410 measured reflections	*l* = −14→11
2949 independent reflections	

### Refinement

**Table:**

Refinement on *F*^2^	Secondary atom site location: difference Fourier map
Least-squares matrix: full	Hydrogen site location: inferred from neighbouring sites
*R*[*F*^2^ > 2σ(*F*^2^)] = 0.051	H-atom parameters constrained
*wR*(*F*^2^) = 0.145	*w* = 1/[σ^2^(*F*_o_^2^) + (0.089*P*)^2^ + 0.36*P*] where *P* = (*F*_o_^2^ + 2*F*_c_^2^)/3
*S* = 1.00	(Δ/σ)_max_ = 0.005
2949 reflections	Δρ_max_ = 0.22 e Å^−^^3^
199 parameters	Δρ_min_ = −0.28 e Å^−^^3^
Primary atom site location: structure-invariant direct methods	Extinction correction: none

### Special details

**Table:**

Geometry. All e.s.d.'s (except the e.s.d. in the dihedral angle between two l.s. planes) are estimated using the full covariance matrix. The cell e.s.d.'s are taken into account individually in the estimation of e.s.d.'s in distances, angles and torsion angles; correlations between e.s.d.'s in cell parameters are only used when they are defined by crystal symmetry. An approximate (isotropic) treatment of cell e.s.d.'s is used for estimating e.s.d.'s involving l.s. planes.
Refinement. Refinement of *F*^2^ against ALL reflections. The weighted *R*-factor *wR* and goodness of fit *S* are based on *F*^2^, conventional *R*-factors *R* are based on *F*, with *F* set to zero for negative *F*^2^. The threshold expression of *F*^2^ > σ(*F*^2^) is used only for calculating *R*-factors(gt) *etc*. and is not relevant to the choice of reflections for refinement. *R*-factors based on *F*^2^ are statistically about twice as large as those based on *F*, and *R*- factors based on ALL data will be even larger.

### Fractional atomic coordinates and isotropic or equivalent isotropic displacement parameters (Å^2^)

**Table:**

	*x*	*y*	*z*	*U*_iso_*/*U*_eq_	
O1	0.33297 (10)	0.27884 (13)	0.71053 (11)	0.0283 (3)	
O2	0.42529 (10)	0.55360 (12)	0.75700 (12)	0.0290 (3)	
O3	0.60516 (11)	0.50766 (12)	0.90418 (11)	0.0291 (3)	
N1	0.07416 (11)	0.46239 (13)	0.28513 (13)	0.0202 (3)	
N2	0.23340 (11)	0.35402 (13)	0.50283 (13)	0.0201 (3)	
H2N	0.2460	0.3835	0.4381	0.024*	
N3	0.52329 (11)	0.49852 (13)	0.79419 (13)	0.0213 (3)	
C2	−0.00265 (14)	0.52243 (15)	0.17885 (15)	0.0212 (3)	
H2	0.0284	0.5671	0.1278	0.025*	
C3	−0.12782 (14)	0.52428 (15)	0.13644 (16)	0.0223 (4)	
H3	−0.1791	0.5691	0.0590	0.027*	
C4	−0.17449 (14)	0.46062 (15)	0.20824 (16)	0.0221 (4)	
H4	−0.2588	0.4591	0.1803	0.027*	
C5	−0.13518 (14)	0.33419 (15)	0.40878 (16)	0.0210 (3)	
H5	−0.2184	0.3298	0.3865	0.025*	
C6	−0.05278 (14)	0.27986 (15)	0.52209 (16)	0.0215 (3)	
H6	−0.0800	0.2388	0.5783	0.026*	
C7	0.07210 (14)	0.28306 (15)	0.55819 (16)	0.0205 (3)	
H7	0.1275	0.2441	0.6373	0.025*	
C8	0.11263 (13)	0.34276 (14)	0.47822 (15)	0.0187 (3)	
C9	0.02839 (13)	0.40129 (14)	0.35897 (15)	0.0181 (3)	
C10	−0.09602 (13)	0.39701 (15)	0.32465 (15)	0.0194 (3)	
C11	0.33334 (13)	0.32539 (15)	0.61325 (15)	0.0197 (3)	
C12	0.44935 (13)	0.34895 (15)	0.60488 (14)	0.0184 (3)	
C13	0.54399 (13)	0.41992 (15)	0.69958 (15)	0.0189 (3)	
C14	0.65666 (14)	0.42618 (15)	0.70338 (16)	0.0220 (3)	
H14	0.7198	0.4741	0.7698	0.026*	
C15	0.67563 (14)	0.36117 (15)	0.60838 (17)	0.0243 (4)	
H15	0.7527	0.3629	0.6100	0.029*	
C16	0.58228 (15)	0.29374 (16)	0.51112 (16)	0.0239 (4)	
H16	0.5951	0.2518	0.4447	0.029*	
C17	0.47000 (14)	0.28663 (15)	0.50942 (15)	0.0216 (3)	
H17	0.4070	0.2389	0.4426	0.026*	

### Atomic displacement parameters (Å^2^)

**Table:**

	*U*^11^	*U*^22^	*U*^33^	*U*^12^	*U*^13^	*U*^23^
O1	0.0213 (6)	0.0419 (7)	0.0199 (6)	−0.0011 (5)	0.0074 (5)	0.0064 (5)
O2	0.0197 (6)	0.0347 (7)	0.0308 (7)	0.0053 (5)	0.0098 (5)	−0.0042 (5)
O3	0.0255 (6)	0.0327 (7)	0.0207 (6)	−0.0014 (5)	0.0026 (5)	−0.0051 (5)
N1	0.0183 (6)	0.0203 (6)	0.0204 (7)	−0.0013 (5)	0.0073 (5)	0.0005 (5)
N2	0.0168 (6)	0.0242 (7)	0.0187 (7)	−0.0022 (5)	0.0074 (5)	0.0018 (5)
N3	0.0191 (6)	0.0210 (7)	0.0213 (7)	−0.0014 (5)	0.0067 (6)	−0.0016 (5)
C2	0.0218 (8)	0.0205 (7)	0.0200 (8)	−0.0014 (6)	0.0081 (6)	0.0023 (6)
C3	0.0201 (7)	0.0195 (7)	0.0229 (8)	0.0002 (6)	0.0056 (6)	0.0004 (6)
C4	0.0174 (7)	0.0197 (7)	0.0267 (9)	0.0012 (6)	0.0076 (6)	−0.0010 (6)
C5	0.0181 (7)	0.0197 (7)	0.0271 (8)	−0.0013 (6)	0.0118 (6)	−0.0031 (6)
C6	0.0244 (8)	0.0186 (7)	0.0259 (9)	−0.0027 (6)	0.0151 (7)	−0.0014 (6)
C7	0.0213 (8)	0.0188 (7)	0.0211 (8)	−0.0005 (6)	0.0092 (6)	−0.0016 (6)
C8	0.0169 (7)	0.0167 (7)	0.0216 (8)	−0.0023 (5)	0.0079 (6)	−0.0034 (6)
C9	0.0184 (7)	0.0156 (7)	0.0196 (8)	−0.0014 (5)	0.0078 (6)	−0.0024 (6)
C10	0.0184 (7)	0.0165 (7)	0.0219 (8)	−0.0010 (5)	0.0077 (6)	−0.0039 (6)
C11	0.0193 (7)	0.0199 (7)	0.0188 (8)	−0.0008 (6)	0.0076 (6)	−0.0015 (6)
C12	0.0171 (7)	0.0189 (7)	0.0165 (7)	0.0014 (5)	0.0050 (6)	0.0040 (5)
C13	0.0188 (7)	0.0181 (7)	0.0180 (8)	0.0014 (5)	0.0066 (6)	0.0013 (6)
C14	0.0165 (7)	0.0205 (7)	0.0259 (8)	−0.0006 (6)	0.0065 (6)	0.0007 (6)
C15	0.0216 (8)	0.0207 (7)	0.0328 (9)	0.0030 (6)	0.0140 (7)	0.0049 (7)
C16	0.0281 (8)	0.0219 (8)	0.0249 (9)	0.0015 (6)	0.0148 (7)	0.0021 (6)
C17	0.0225 (8)	0.0223 (8)	0.0189 (8)	−0.0012 (6)	0.0081 (6)	0.0009 (6)

### Geometric parameters (Å, °)

**Table:**

O1—C11	1.219 (2)	C6—C7	1.417 (2)
O2—N3	1.2307 (17)	C6—H6	0.95
O3—N3	1.2257 (17)	C7—C8	1.373 (2)
N1—C2	1.320 (2)	C7—H7	0.95
N1—C9	1.365 (2)	C8—C9	1.434 (2)
N2—C11	1.3562 (19)	C9—C10	1.416 (2)
N2—C8	1.4028 (19)	C11—C12	1.507 (2)
N2—H2N	0.88	C12—C17	1.389 (2)
N3—C13	1.463 (2)	C12—C13	1.396 (2)
C2—C3	1.408 (2)	C13—C14	1.383 (2)
C2—H2	0.95	C14—C15	1.386 (2)
C3—C4	1.366 (2)	C14—H14	0.95
C3—H3	0.95	C15—C16	1.383 (2)
C4—C10	1.415 (2)	C15—H15	0.95
C4—H4	0.95	C16—C17	1.389 (2)
C5—C6	1.366 (2)	C16—H16	0.95
C5—C10	1.416 (2)	C17—H17	0.95
C5—H5	0.95		
			
C2—N1—C9	117.28 (13)	N1—C9—C10	123.11 (14)
C11—N2—C8	128.52 (13)	N1—C9—C8	117.16 (13)
C11—N2—H2N	115.7	C10—C9—C8	119.70 (14)
C8—N2—H2N	115.7	C4—C10—C5	123.56 (14)
O3—N3—O2	124.26 (14)	C4—C10—C9	117.17 (14)
O3—N3—C13	118.14 (13)	C5—C10—C9	119.23 (14)
O2—N3—C13	117.59 (13)	O1—C11—N2	124.71 (14)
N1—C2—C3	123.99 (15)	O1—C11—C12	121.09 (14)
N1—C2—H2	118.0	N2—C11—C12	114.12 (13)
C3—C2—H2	118.0	C17—C12—C13	117.75 (14)
C4—C3—C2	119.02 (15)	C17—C12—C11	119.90 (13)
C4—C3—H3	120.5	C13—C12—C11	121.93 (14)
C2—C3—H3	120.5	C14—C13—C12	122.48 (15)
C3—C4—C10	119.39 (14)	C14—C13—N3	117.59 (13)
C3—C4—H4	120.3	C12—C13—N3	119.80 (13)
C10—C4—H4	120.3	C13—C14—C15	118.66 (14)
C6—C5—C10	119.76 (14)	C13—C14—H14	120.7
C6—C5—H5	120.1	C15—C14—H14	120.7
C10—C5—H5	120.1	C16—C15—C14	119.96 (15)
C5—C6—C7	121.88 (15)	C16—C15—H15	120.0
C5—C6—H6	119.1	C14—C15—H15	120.0
C7—C6—H6	119.1	C15—C16—C17	120.81 (15)
C8—C7—C6	119.64 (15)	C15—C16—H16	119.6
C8—C7—H7	120.2	C17—C16—H16	119.6
C6—C7—H7	120.2	C12—C17—C16	120.29 (15)
C7—C8—N2	125.45 (14)	C12—C17—H17	119.9
C7—C8—C9	119.78 (14)	C16—C17—H17	119.9
N2—C8—C9	114.76 (13)		
			
C9—N1—C2—C3	−1.5 (2)	C8—C9—C10—C5	−0.3 (2)
N1—C2—C3—C4	0.0 (2)	C8—N2—C11—O1	3.0 (3)
C2—C3—C4—C10	1.4 (2)	C8—N2—C11—C12	179.71 (14)
C10—C5—C6—C7	−0.7 (2)	O1—C11—C12—C17	120.18 (17)
C5—C6—C7—C8	0.4 (2)	N2—C11—C12—C17	−56.67 (19)
C6—C7—C8—N2	179.31 (14)	O1—C11—C12—C13	−52.2 (2)
C6—C7—C8—C9	0.0 (2)	N2—C11—C12—C13	130.96 (15)
C11—N2—C8—C7	−9.2 (3)	C17—C12—C13—C14	−2.1 (2)
C11—N2—C8—C9	170.17 (14)	C11—C12—C13—C14	170.39 (14)
C2—N1—C9—C10	1.7 (2)	C17—C12—C13—N3	173.65 (13)
C2—N1—C9—C8	−176.46 (14)	C11—C12—C13—N3	−13.8 (2)
C7—C8—C9—N1	178.25 (13)	O3—N3—C13—C14	−37.9 (2)
N2—C8—C9—N1	−1.14 (19)	O2—N3—C13—C14	140.59 (15)
C7—C8—C9—C10	0.0 (2)	O3—N3—C13—C12	146.16 (15)
N2—C8—C9—C10	−179.40 (13)	O2—N3—C13—C12	−35.4 (2)
C3—C4—C10—C5	176.77 (14)	C12—C13—C14—C15	1.0 (2)
C3—C4—C10—C9	−1.2 (2)	N3—C13—C14—C15	−174.87 (13)
C6—C5—C10—C4	−177.25 (15)	C13—C14—C15—C16	1.1 (2)
C6—C5—C10—C9	0.6 (2)	C14—C15—C16—C17	−2.0 (2)
N1—C9—C10—C4	−0.4 (2)	C13—C12—C17—C16	1.2 (2)
C8—C9—C10—C4	177.72 (13)	C11—C12—C17—C16	−171.51 (14)
N1—C9—C10—C5	−178.44 (13)	C15—C16—C17—C12	0.9 (2)

### Hydrogen-bond geometry (Å, °)

**Table:**

*D*—H···*A*	*D*—H	H···*A*	*D*···*A*	*D*—H···*A*
C3—H3···O3^i^	0.95	2.55	3.209 (2)	127
C4—H4···O2^ii^	0.95	2.48	3.319 (2)	147
C17—H17···O1^iii^	0.95	2.42	3.160 (2)	135

Symmetry codes: (i) *x*−1, *y*, *z*−1; (ii) −*x*, −*y*+1, −*z*+1; (iii) *x*, −*y*+1/2, *z*−1/2.
